# Species- and developmental stage-specific effects of allelopathy and competition of invasive *Impatiens glandulifera* on co-occurring plants

**DOI:** 10.1371/journal.pone.0205843

**Published:** 2018-11-07

**Authors:** Judith Bieberich, Marianne Lauerer, Maria Drachsler, Julian Heinrichs, Stefanie Müller, Heike Feldhaar

**Affiliations:** 1 Ecological Botanical Gardens, BayCEER (Bayreuth Center for Ecology and Environmental Research), University of Bayreuth, Bayreuth, Germany; 2 Department of Animal Ecology 1, BayCEER (Bayreuth Center for Ecology and Environmental Research), University of Bayreuth, Bayreuth, Germany; Estacion Experimental del Zaidin, SPAIN

## Abstract

**Background:**

Impacts of invasive species on native communities are often difficult to assess, because they depend on a range of factors, such as species identity and traits. Such context-dependencies are poorly understood yet, but knowledge is required to predict the impact of invasions.

**Materials and methods:**

We assessed species- and developmental stage-specificity of competitive and allelopathic effects of the invasive plant *Impatiens glandulifera* on different developmental stages of four native plant species. While some studies have shown a reduction in plant growth caused by *I*. *glandulifera*, the magnitude of its impact is ambiguous. For our study we used seedlings and juveniles of *I*. *glandulifera* and the native target species *Geum urbanum*, *Filipendula ulmaria*, *Urtica dioica*, and *Salix fragilis* (seedlings only of the latter), which often co-occur with *I*. *glandulifera* in different habitats. Plants were grown in competition with *I*. *glandulifera* or treated with *I*. *glandulifera* leaf material, or 2-metoxy-1,4-naphtoquinone (2-MNQ), its supposedly main allelochemical.

**Results and conclusions:**

Overall *I*. *glandulifera* had a negative effect on the growth of all target species depending on the species and on the plant’s developmental stage. *F*. *ulmaria* was the least affected and *U*. *dioica* the most, and seedlings were less affected than juveniles. The species-specific response to *I*. *glandulifera* may lead to an altered community composition in the field, while growth reduction of seedlings and juveniles should give *I*. *glandulifera* an advantage in cases where plant recruitment is crucial. 2-MNQ led to minor reductions in plant growth, suggesting that it may not be the only allelopathic substance of *I*. *glandulifera*. Surprisingly, *I*. *glandulifera* was not fully tolerant to 2-MNQ. This autotoxicity could contribute to *I*. *glandulifera* population dynamics. We conclude that *I*. *glandulifera* reduces the growth of native vegetation and alters early successional stages without fully hindering it.

## Introduction

Invasive species are considered to be among the most important drivers of biodiversity loss worldwide [1]. They affect native ecosystems negatively in many ways. They can suppress growth of native species and alter ecosystem processes and structures [2,3]. However, it is difficult to comprehensively assess the impact of invasive species due to context-dependencies. The outcome of an invasion is influenced by the invaded ecosystem, invasion stage and species traits [4]. Depending on the invaded ecosystem the invasive species interacts with different native species. Basically, different species should react differently to the invasion and the interaction between native and invasive plants could depend on their developmental stage. Such developmental-stage specific interactions are rarely studied but such knowledge would improve our ability to understand and predict the overall effect of a particular invasive species as well as invasion processes in general.

The plant genus *Impatiens* is an ideal model taxon for the study of context-dependencies [4] such as species- and developmental stage specificity. Several species of this genus are widely introduced and constitute a broad range of invasiveness. In Central Europe *Impatiens glandulifera* Royle is one of the most famous alien plants with its strikingly tall growth of more than 2 m height and its large, purple flowering stands [5]. Introduced to England in the 19^th^ century, it has spread over nearly the whole of Europe and is nowadays very common [6–9]. It mainly followed river systems but subsequently also invaded sites at a distance from the rivers [8]. Invaded habitats are riparian sites, mesotrophic grasslands and woodlands, semi-natural sites but also forests out of the riparian zone [5,7,9,10]. Generally *I*. *glandulifera* is favored by disturbances [5,11]. In 2017 *I*. *glandulifera* was added to the list of invasive alien species of Union concern [12,13] However, the degree of invasiveness is perceived differently in different countries. In 2014 it was included in the black list of plants evidentially harming native biodiversity in Switzerland [14]. In contrast German nature conservation authorities rate *I*. *glandulifera* as potentially invasive, with an assumed threat to native species [15]. This moderate ranking was justified with the mixed results from field studies on the impact of *I*. *glandulifera* on native plant communities [11,16–19]. Thus, a deeper understanding of the interaction between *I*. *glandulifera* and its co-occurring plant species is required.

Possible mechanisms for the suppression of co-occurring plants are allelopathy and competition [20]. *I*. *glandulifera* produces 2-methoxy-1,4-naphthoquinone (2-MNQ), which is considered to be its main allelopathic substance. [21–25]. 2-MNQ gets rinsed off the leaves by rainwater, is present in the soil and inhibits mycorrhiza growth [21]. *I*. *glandulifera* litter leachates and plant material extracts reduce the germination of other species such as *Leucosinapis alba* [26] or *Scrophularia nodosa* [21], with the concentration of 2-MNQ in the extracts correlating with their inhibitory effect [21]. *I*. *glandulifera* litter reduces seed germination species-specifically [27], and tree saplings suffer in invaded field sites [28,29]. It is also a strong competitor of *Urtica dioica* [30] and conspecifics as *Impatiens noli-tangere* [31,32]. In contrast, other studies did not find such negative effects. Thus, seed germination [33], and forest recruitment were not restricted in invaded forest sites [34]. These varied outcomes may be caused by context-dependencies, because all these studies were conducted with different settings regarding study conditions, target plants, their developmental stages and the parts of *I*. *glandulifera* plants considered.

Native species co-occrring with *I*. *glandulifera* are expected to differ in their susceptibility to the invasive plant, due to differences in their traits and autecology. Additionally, life stages are known to affect interactions between species [35]. Thus, seedlings could respond differently to *I*. *glandulifera* than juvenile plants. Both developmental stages are important for the recruitment of plants and a negative impact on either of the stages or both may result in altered plant community composition. Overall, the direct role of 2-MNQ in mediating plant-plant interactions is still unclear, including its effect on *I*. *glandulifera* itself. If *I*. *glandulifera* benefits from inhibiting growth of co-occurring plants via 2-MNQ it should be less sensitive to 2-MNQ than native plants in order to have an advantage by suppressing growth of co-occurring plants. To comprehensively explore species- and developmental stage specific effects and the mechanisms of the impact of the invasive *I*. *glandulifera* we investigated competitive and allelopathic effects on different co-occurring native species. Here, we asked the following questions: 1) Are competitive and allelopathic effects species-specific? 2) Do these effects depend on plant developmental stage, in particular do the effects on seedlings and juveniles differ? 3) Is 2-MNQ the substance responsible for the allelopathic effects of *I*. *glandulifera*? 4) Is *I*. *glandulifera* tolerant to its own chemical weapons? To answer these questions we experimentally tested the effect of competition by *I*. *glandulifera* as well as leaf material and pure 2-MNQ on the growth of seedlings and juveniles of selected native species that co-occur with *I*. *glandulifera* in different habitats.

## Materials and methods

### Plant species

As native target species species we used plant species that regularly co-occur with *I*. *glandulifera* in riparian habitats or deciduous woodlands in Germany: *Filipendula ulmaria* (L.) Maxim. is common in tall herbaceous vegetation of elder woods and meadows, *Geum urbanum* L. in woodlands and disturbed habitats. *Urtica dioica* L. is typical for tall herbaceous vegetation especially in nutrient rich sites [36,37]. All target species are perennial and can form dominant stands. We therefore expected that they should cope with competition by *I*. *glandulifera* relatively well. In the seedling trial *Salix fragilis* L., a tree from wetlands and early successional stages at riversides [38], was used additionally, as well as *Lepidium sativum* L., a control species not co-occrring with *I*. *glanduifera* but often used in allelopathy experiments [39]. *I*. *glandulifera* was used as target species and to test its impact on other plants. No permission was required to use this invasive plant species because all trials were conducted before it was included in the list of invasive alien species of Union concern [12,13]. Flowers of *I*. *glandulifera* juveniles were removed prior to seed set and all its plant material was destroyed after the trials.

### Seedling trials

Seeds of all species except for *L*. *sativum* were collected in 3–8 field sites per species in the region of Bayreuth (Germany). We were permitted by the government of Upper Franconia (Regierung von Oberfranken) to collect plant material in this region. Neither one of the sites nor one of the species is under nature protection. In these sites, *I*. *glandulifera* was mostly absent, except for a few occasions where it was moderately intermixed with the native vegetation. Seeds of each species were pooled for the experiment. Seeds of *I*. *glandulifera*, *F*. *ulmaria*, *U*. *dioica*, *G*. *urbanum* were collected in autumn 2014 from a minimum of 20 plants per site. They were dry stored under refrigeration (8 °C). Seeds of *S*. *fragilis* were collected in early June from 3 sites and 3–6 trees per site, mixed with the hybrid *Salix x rubens* Schrank. These seeds were stored under refrigeration (4 °C). Seeds of *L*. *sativum* were commercially obtained (Kiepenkerl, article number 2498, year 2014/2015). To overcome dormancy in seeds they were warm–cold stratified within wet quartz sand, *G*. *urbanum* and *F*. *ulmaria* (2 weeks at 30 °C and 4–11 weeks at 4 °C) and seeds of *I*. *glandulifera* (10–12 weeks at 4 °C). The trials with seedlings were conducted from beginning of June (when *S*. *fragilis* fructified in this particular year) to August 2015. Seeds of all species were sown every couple of days as required to obtain as many germinating seeds of the different species at the same time. They were placed on wet filter paper in petri dishes close to window exposed to natural light at room temperature and kept moist with a fungicide solution (Previcur N 1.5 ml / 1 l water; Bayer). As soon as radicles emerged (one day to several weeks, depending on the species) the germinated seeds were used for the trials. Maximum length of the radicle was 4 mm for *I*. *glandulifera* and 3 mm for all other species chosen for trials.

**Fig 1:**
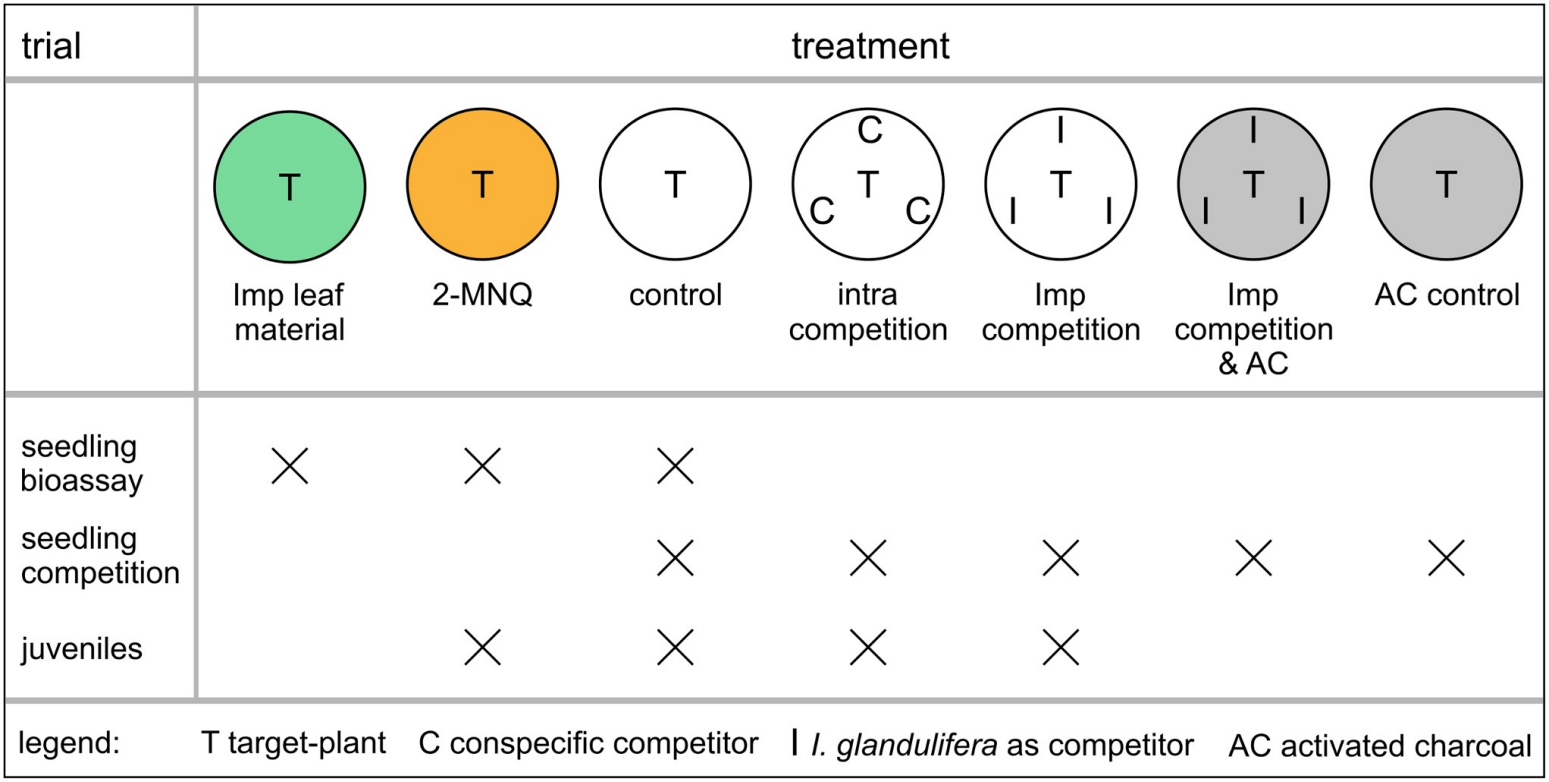
Treatments applied in the seedling bioassay, seedling competition and the juveniles trial. Target plants were grown solitarily (control) or in intraspecific competition with their conspecifics (intra competition) or with *I*. *glandulifera* (Imp competition). The treatments are named as in all other figures. If the target plant is *I*. *glandulifera* intraspecific competition and competition with *I*. *glandulifera* is notably one and the same. Coloration indicates a treatment with *I*. *glandulifera* leaf material (green), pure 2-MNQ (orange) or activated charcoal (AC, grey). In the seedling bioassay and seedling competition trials plants were grown on agar in microwell plates, juveniles were grown in soil in pots.

To test the competitive and allelopathic effects of *I*. *glandulifera* seedlings, we grew the target seedlings on agar (0.5% w/v) either solitarily (control), surrounded by three conspecific seedlings (intraspecific competition), or in competition with three *I*. *glandulifera* seedlings (Fig 1). Hereafter, the plant that is subjected to the treatments is defined as target plant or target seedling. To distinguish between a growth reduction due to an allelopathic or a competitive effect we additionally added activated charcoal (0.05% w/v) to the agar. The activated charcoal is expected to absorb allelopathic substances potentially released by *I*. *glandulifera* seedlings [40]. To control for general impacts of the activated charcoal on the seedlings’ growth we included a treatment with one single target seedling on agar containing just the activated charcoal. The five treatments were randomly assigned to the wells of a 6-microwell plate (Nunc^™^, Thermo Fischer Scientific, 9.6 cm^2^ per well). The wells were filled with 5 ml of the appropriate agar and the germinated seeds were placed in five wells on the solidified agar with one blank. We conducted the experiment with 20 replicates per species, except for *S*. *fragilis* where we were only able to obtain 11 replicates. The seedlings were grown in a climate chamber (25 °C, 70% humidity, 12/12 h light/dark cycle, light source Osram Lumilux HO 80W/840) for six days. After this the target seedlings were removed from the agar and their root length was measured to the nearest 0.1 mm with a digital caliper. In the case of branched roots, which was often observed in *I*. *glandulifera*, the length of the longest branch was measured. Then the entire seedling was dried for 24 h at 60 °C and weighed to the nearest 1 μg (Santorius micro weighing scale M 500 P). In 2015 the germination rate of *F*. *ulmaria* was unfortunately so low that the competition trial could not be conducted for this species. Therefore, it was performed in June to July 2017 with 13 replicates but using the seeds collected in 2015 for the juvenile trial (see below).

To test if 2-MNQ is responsible for an allelopathic impact of *I*. *glandulifera* a bioassay was conducted on agar (derived from [41]), treating seedlings of the target species with pure 2-MNQ and with leaf material of *I*. *glandulifera* seedlings (Fig 1). Leaf material of *I*. *glandulifera* seedlings was used as it was shown that it contains high concentrations of 2-MNQ and that its extracts inhibit seed germination [21]. *I*. *glandulifera* seedlings whose primary leaves were still shorter than the cotyledons (average length of the cotyledons 18 ± 3 mm, n = 20) were collected in the end of April 2015 at four sites in Bayreuth comprising of habitats such as forest, riparian forest and wet meadow. Cotyledons and primary leaves were dried for 24 h at 70 °C and ground with a pestle. This powder was added to fresh agar (60 °C; 0.5% w/v) at the concentrations of 0, 0.15, 0.30, 0.60, 1.20 and 2.40 g/l. In a second approach, 2-MNQ (Sigma-Aldrich) was used. As the solid 2-MNQ is not solvable in water it was solved in ethanol (2 mg 2-MNQ per 1 ml 80% ethanol) and the solvent was added to the agar to obtain the final concentrations of 0, 0.8, 1.6, 3.2, 6.4 and 12.8 mg 2-MNQ per liter agar. For the control without 2-MNQ, 6.4 μl 80% ethanol was added per 1 ml agar as this is the highest used amount of ethanol solution. The concentrations of *I*. *glandulifera* leaf material and 2-MNQ used were chosen according to the study of [21]. Leaf extracts are known to reduce seed germination and the low 2-MNQ concentrations are known to affect mycorrhiza growth while the high 2-MNQ concentrations were found in rainwater rinsed from *I*. *glandulifera* plants. In total there were 12 different treatments (5 concentrations of *I*. *glandulifera* leaf material plus control and 5 concentrations of 2-MNQ plus control). The treatments were randomly assigned to the wells of a 24-microwell plate (Nunc^™^, Thermo Fischer Scientific, 1.8 cm^2^ per well), with two replicates per treatment resulting in a block design. Each well was filled with 1 ml appropriate agar. Finally, per well one germinated seed was put on the solidified agar. If fewer than 24 germinated seeds were available at once, only one replicate per treatment was realized within one particular plate. For *I*. *glandulifera*, *L*. *sativum*, *U*. *dioica*, *G*. *urbanum* and *S*. *fragilis* in total 12 replicates per treatment were performed. For *F*. *ulmaria* which germinated rather poorly, we had only seven replicates. The seedlings were grown at the same time and in the same climate chamber as those of the experiment on seedling competition. The positions of all plates within the climate chamber were changed randomly each day. Same as in the seedling competition experiment, the seedlings’ root length and dry biomass was measured after 6 days of growth.

### Juvenile trial

Competition and impact of 2-MNQ was studied for the first-year growth of *F*. *ulmaria*, *U*. *dioica*, *G*. *urbanum* and on *I*. *glandulifera*. Seeds were collected in 2015, stored and stratified as they were in 2014 for the seedling trials but *I*. *glandulifera* did not need stratification. Seeds were sown in the first and second week of April 2016 on potting compost in sowing shells which were placed in a greenhouse (17–27 °C). After 2 weeks the seedlings were pricked out to pots with a volume of 230 cm^3^ soil within QuickPot trays. Two weeks after pricking *I*. *glandulifera* plants were brought outdoors. Four weeks after pricking (third and fourth week of May) plants of medium and homogeneous size were used for the trial.

The individual plants as well as the processing order were randomly assigned to the 4 treatments, each in 10 repetitions. The target plants were potted in 20-liter pots according to the treatments, either solitarily, in intraspecific competition or in competition with *I*. *glandulifera* (Fig 1). If *I*. *glandulifera* is the target species intraspecific competition and competition with *I*. *glandulifera* is notably one and the same, resulting in three instead of four treatments overall. For the 2-MNQ treatment 1 liter of a 10 mg/l 2-MNQ solution was applied to a single target plant following regular watering. For this purpose, each time 2-MNQ (Sigma Aldrich) was dissolved in pure ethanol (2 mg/ml) and diluted with tap water. The potting soil contained 39% white peat, 11% black peat, 20% coconut fibre, 15% lava granules and 15% bark compost. Per 1 m^3^ the substrate was fertilized with 3 kg slow-release fertilizer with macro-nutrients (Osmocote Exact Protect 14% N, 8% P_2_O_5_, 11% K_2_O, 2% MgO, 8−9 month effect duration; EVERRIS) and 200 g slow-release fertilizer with micro-nutrients (Radigen 2% Fe, 1.5% Cu, 1% Mn, 0.8% Mo, 0.6% B, 0.5% Zn; TERRAFLOR) and 1 kg carbonic agricultural lime. At the time of potting target plants of *I*. *glandulifera* were 19 ± 4 cm (n = 30; ten repetitions per three treatments) in height, *U*. *dioica* 19 ± 6 cm, *G*. *urbanum* 7 ± 2 cm and *F*. *ulmaria* 7 ± 2 cm (each n = 40; ten repetitions per four treatments). Pots were placed within 5 blocks of 30 pots outdoors in the Ecological–Botanical Gardens of the University of Bayreuth, Germany. Each block contained two replicates of all treatments and all species randomly assigned to the positions in the blocks. The substrate was always kept moist by watering or natural precipitation. During the trial air temperature was 20 °C in average (min 7 °C, max 40 °C) and humidity 75% (min 21%, max 100%), both measured hourly using an iButton (DS1923, Maxim).

Ten weeks after potting (fourth week of July and first week of August) the growth of the target plants was quantified. Of the stem building species *I*. *glandulifera* and *U*. *dioica* height (from soil to the highest point of the plant) was measured with a folding ruler to the nearest 0.5 cm. For the rosette forming species *G*. *urbanum* and *F*. *ulmaria* the rosette’s projection area was approximated, assuming the rosette to be an ellipse: we measured the widest expansion of the rosette and its orthogonal expansion with a folding ruler to the nearest 0.5 cm as axes for calculation of the area of ellipse. Of all species the above-ground biomass was harvested, dried at 90 °C for two days and weighed to the nearest 0.01 g with a weighing scale (Mettler PM 4600).

### Statistical analyses

Data analyses were done using the software package R [42], RSTUDIO 99.9.9 and various additional packages: LME4 [43], GGPLOT2 [44], PLYR [45], MULTCOMP [46], COWPLOT [47], R COLORBREWER [48], BROOM [49] and RMISC [50]. Figures were arranged with INKSCAPE 0.92. In the seedling trials some of the germinated seeds died shortly after they were placed on the agar; there was no visible root elongation and the cotyledons did not emerge from the testa. In total there were 7 dead seedlings in the trial on competition and allelopathy, 6 in the bioassay with 2-MNQ and 17 in the bioassay with leaf material. The count did not depend on the treatment, except the bioassay with leaf material (chi-squared test χ^2^ = 13.27, df = 5, *p* = 0.021). When mortality was analyzed per species this was not significant in any case. Thus, we consider death of seedlings to be a transplantat effect and excluded them from growth analyses. Seedlings were also excluded from analyses if they were conspicuously infested by fungi (30 of 474 seedlings in the trial on competition and allelopathy, 38 of 804 in the bioassays), or if, less than three competitor-seedlings had grown. This led to varying sample sizes within a species. To analyze the growth of the target plants linear mixed effect models were used with the microwell-plate (seedlings) or block (juveniles) as random factor. The models were built with the lmer function of lme4-package with a random intercept error term. The full models were compared against null-models with likelihood ratio tests (anova function), resulting χ^2^-values, degrees of freedom and p-values give the significance of the models and were reported.

First, with the log-transformed data it was tested whether the growth depended on plant species, treatment and their interaction. Separate *p*-values for the single predictors were calculated using the CAR-package [51]. In a second step differences in growth between treatments were tested for each species separately with a linear mixed effect model and a post-hoc Tukey’s HSD test. Because of heteroscedasticity, biomass, rosette projection area and growth height of juveniles were log-transformed. In the seedling bioassay, it was tested whether growth declined exponentially with increasing concentration of 2-MNQ or *I*. *glandulifera* leaf material respectively. Therefore the regression equation f(x) = exp(*a*x + *b*) was fitted. To compare the impact of competition and *I*. *glandulifera* allelochemicals, between both developmental stages and the species *G*. *urbanum*, *F*. *ulmaria*, *U*. *dioica* and *I*. *glandulifera* a relative interaction index [30,52] was calculated as, comparing a certain treatment with the related control (mean(treatment)–mean(control) / mean(treatment)+mean(control)). The resulting values were visualized in a heatmap.

## Results

### Seedling competition and allelopathy via roots

**Fig 2:**
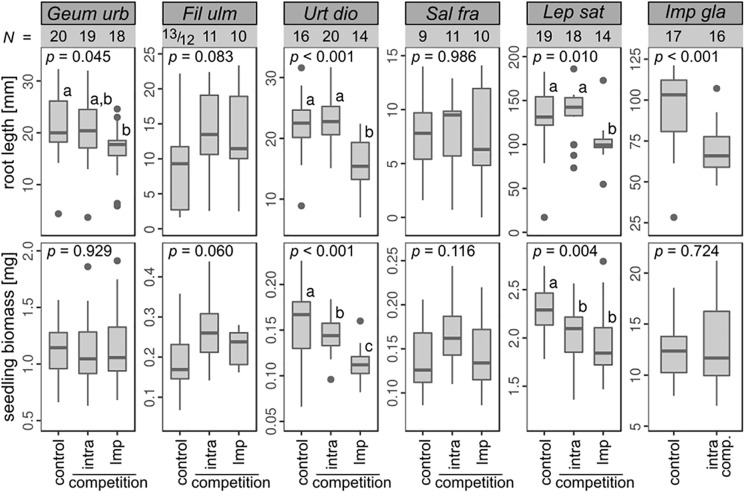
Effect of competition on root length and total dry biomass of seedlings. Seedlings of the target species *Geum urbanum* (*Geum urb*), *Filipendula ulmaria* (*Fil ulm*), *Urtica dioica* (*Urt dio*), *Salix fragilis* (*Sal fra*), *Lepidium sativum* (*Lep sat*) and *Impatiens glanduifera* (*Imp gla*) were grown solitarily (control), in intraspecific competition (intra) or in competition with *Impatiens glandulifera* seedlings (Imp). Note that the scale of the y-axis varies among species. Number of observations (*N*) are shown. It was tested if the growth depended on the treatments using a linear mixed effect model (microwell plate in which the seedlings were grown as random factor) (*p*-values are given); different letters mark significant differences among treatments (post-hoc Tukey’s HSD test for *p* < 0.050). Boxes represent the first and third quartiles, bands inside the boxes the median. Whiskers are restricted to the 1.5 interquartile ranges. Datapoints not included in the whiskers are depicted as dots.

In the seedling competition trial, we grew the target seedlings solitarily, in intraspecific competition and in competition with *I*. *glandulifera*. An overall linear mixed-effect model (χ^2^ = 295.77, df = 16, *N* = 255, *p* < 0.001) showed that the root length of the seedlings depended on the species (χ^2^ = 1173.81, df = 5, *p* < 0.001), the treatment (χ^2^ = 9.87, df = 2, *p* = 0.007) and the interaction between species and treatment (χ^2^ = 17.32, df = 9, *p* = 0.044). This means that species responded differently to the treatments. In the control treatments (Fig 2) median root length varied from 7.8 mm (*S*. *fragilis*) to 131 mm (*L*. *sativum*) and the biomass from 0.1 mg (*S fragilis*) to 12.4 mg (*I*. *glandulifera*). Competition affected the root length of *G*. *urbanum* (χ^2^ = 6.22, df = 2, *p* = 0.045), *U*. *dioica* (χ^2^ = 18.09, df = 2, *p* < 0.001), *L*. *sativum* (χ^2^ = 9.16, df = 2, *p* = 0.010) and *I*. *glandulifera* (χ^2^ = 11.06, df = 1, *p* < 0.001) as well as seedling biomass of *U*. *dioica* (χ^2^ = 17.98, df = 2, *p* < 0.001) and *L*. *sativum* (χ^2^ = 10.87, df = 2, *p* = 0.004). Compared to the control treatment (solitary seedlings), intraspecific competition (four conspecific seedlings per well) had no impact on the root length of the native target species and *L*. *sativum* but reduced the biomass of *U*. *dioica* and *L*. *sativum*. Also root length of *I*. *glandulifera* in competition with its conspecifics was reduced. Competition with *I*. *glandulifera* seedlings reduced the root length of *G*. *urbanum*, *U*. *dioica* and *L*. *sativum* as well as the biomass of *U*. *dioica* and *L*. *sativum* in comparison to the control treatment. The mean root length of the most affected native species, *U*. *dioica* was 32% and those of the least affected *G*. *urbanum* 13% shorter than in the controls. Interspecific competition with *I*. *glandulifera* had a stronger impact on the growth of seedlings in comparison to intraspecific competition as the root length and biomass of *U*. *dioica* and root length of *L*. *sativum* were reduced more strongly. To investigate whether *I*. *glandulifera* seedlings release allelopathic substances into the agar that are responsible for the growth reduction we added activated charcoal to the agar. In the control treatment it had no negative effect on the growth of a single target seedling. Seedling biomass of *G*. *urbanum* was even slightly enhanced (χ^2^ = 5.35, df = 1, *p* = 0.021, linear mixed effect model). However, in competition with *I*. *glandulifera* the addition of activated charcoal did not improve seedling growth. Root length of *L*. *sativum* (χ^2^ = 11.14, df = 1, *p* < 0.001) and biomass of *U*. *dioica* (χ^2^ = 5.01, df = 1, *p* = 0.025) were even reduced in comparison to the *I*. *glandulifera* competition treatment without activated charcoal (linear mixed-effect models).

### Impact of 2-MNQ and *I*. *glandulifera* leaf material on seedling growth

For the bioassay with 2-MNQ an overall linear mixed-effect model (χ^2^ = 136.64, df = 11, *N* = 392, *p* < 0.001) revealed that the root length depended on species (χ^2^ = 687.49, df = 5, *p* < 0.001) and concentration of 2-MNQ (χ^2^ = 19.28, df = 1, *p* < 0.001), but the interaction term of both was not significant (χ^2^ = 7.11, df = 5, *p* = 0.213). While *G*. *urbanum*, *F*. *ulmaria*, *S fragilis* and *I*. *glandulifera* were not affected by 2-MNQ, the root length of *U*. *dioica* and *L*. *sativum* declined exponentially with increasing concentration of 2-MNQ (Table 1, S1 Fig). However, the correlation coefficient *a* showed only a slight decline (Table 1) and the *R*^*2*^ values of the corresponding linear models without random factor were very low (S1 Fig), showing a weak correlation. The seedling biomass was not affected by 2-MNQ at all.

**Table 1:** Seedlings growth as a function of the concentration of 2-MNQ and *I*. *glandulifera* leaf material.

	species	*N*	root length				seedling biomass			
			χ^2^ DF = 1	*p*-value	regression coefficients		χ^2^ DF = 1	*p*-value	regression coefficients	
					*a*	*b*			*a*	*b*
2-MNQ	*Geum urb*	71	2.00	0.157			0.20	0.653		
	*Fil ulm*	42	0.52	0.470			0.01	0.926		
	*Urt dio*	72	12.36	< 0.001	-0.042	3.174	1.19	0.276		
	*Sal fra*	72	0.18	0.671			1.94	0.164		
	*Lep sat*	72	11.93	0.001	-0.043	4.923	1.52	0.217		
	*Imp gla*	63	3.33	0.068			0.27	0.605		
leaf material	*Geum urb*	71	28.50	< 0.001	-0.376	3.021	0.47	0.492		
	*Fil ulm*	30	3.37	0.066			0.19	0.661		
	*Urt dio*	66	54.72	< 0.001	-0.716	3.012	6.45	0.011	-0.102	-1.946
	*Sal fra*	71	26.25	< 0.001	-0.835	2.277	3.50	0.061		
	*Lep sat*	72	24.58	< 0.001	-0.652	4.730	3.20	0.074		
	*Imp gla*	64	0.05	0.821			0.06	0.801		

For each target species it was tested whether root length and total dry biomass declined exponentially with increasing concentration of 2-MNQ (0–12.8 mg/l) and amount of *I*. *glandulifera* leaf material (0–2.4 g/l) that was added to the agar. See S1 and S2 Figs for plots of the raw data. Using a linear mixed effect model (microwell plate in which the seedlings were grown as random factor) the regression equation f(x) = exp(*a*x + *b*) was fitted; χ^2^-values, resulting *p*-values and, in the case of significance, the regression coefficients *a* and *b* are given. A negative sign of *a* implies a decline of the fitted curve, its absolute value the strength of the decline. The coefficient *b* gives the y-intercept, calculated as exp(b). target species are abbreviated as follows: *Geum urbanum* (*Geum urb*), *Filipendula ulmaria* (*Fil ulm*), *Urtica dioica* (*Urt dio*), *Salix fragilis* (*Sal fra*), *Lepidium sativum* (*Lep sat*) and *Impatiens glanduifera* (*Imp gla*).

For the bioassay with *I*. *glandulifera* leaf material an overall linear mixed-effect model (χ^2^ = 179.62, df = 11, *N* = 374, *p* < 0.001) revealed that the root length depended significantly on plant species (χ^2^ = 125.12, df = 5, *p* < 0.001), concentration of leaf material (χ^2^ = 107.84, df = 1, *p* < 0.001) and likewise their interaction term (χ^2^ = 37.81, df = 5, *p* < 0.001). Thus, species responded differently to *I*. *glandulifera* leaf material. *I*. *glandulifera* leaf material had a higher impact on the seedling growth than 2-MNQ. It reduced the root length of *G*. *urbanum*, *U*. *dioica*, *S fragilis* and *L*. *sativum* (Table 1, S2 Fig). Seedling biomass of *U*. *dioica* slightly declined with increasing concentration of the leaf material (linear mixed-effect model *p* = 0.011, but linear model *R*^2^ = 0.08; Table 1, S2 Fig). The regression coefficient was higher in the bioassay with *I*. *glandulifera* leaf material compared to the one in trials with 2-MNQ (Table 1), e.g. in *U*. *dioica* the regression coefficient of root length with leaf material was -0.716 (median declined from 26 to 5 mm) compared to -0.042 with 2-MNQ (median declined from 26 to 14 mm). *F*. *ulmaria* and *I*. *glandulifera* were not significantly affected at all, but *F*. *ulmaria* root length slightly declined with higher leaf material concentration and *I*. *glandulifera* root length with higher 2-MNQ concentration (Table 1).

### Allelopathy and competition in juveniles

**Fig 3:**
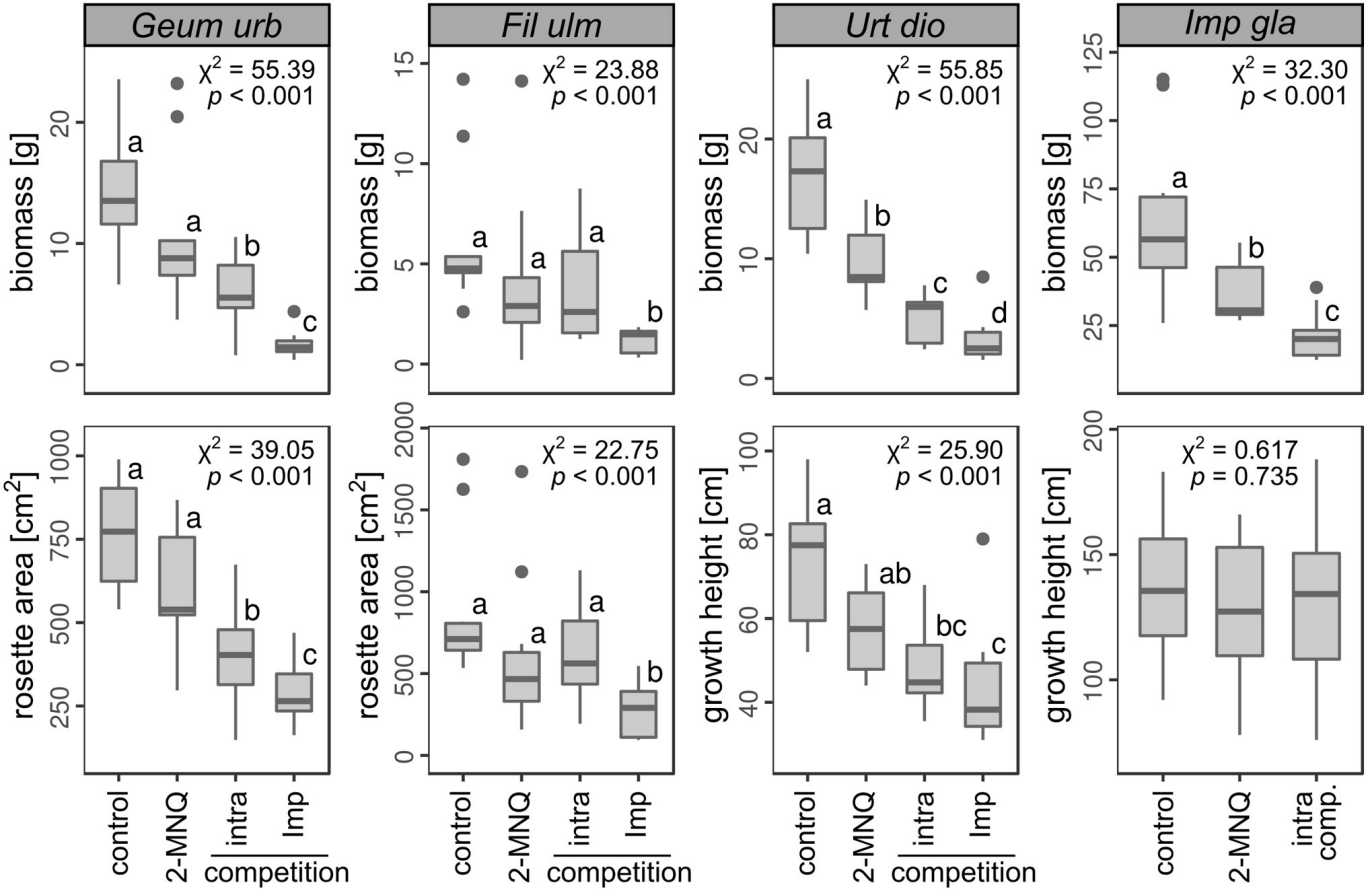
Effect of 2-MNQ and competition on the growth of juvenile target plants. For all target species the aboveground dry biomass after 10 weeks growth in pots is shown. For *G*. *urbanum* and *F*. *ulmaria* also the projection area of the rosettes and for *U*. *dioica* and *I*. *glandulifera* the plant height is shown. The dependence of the growth on the treatments was tested with a linear mixed effect model (*N* = 10 per treatment; block in which the pots were arranged as random factor) using log-transfomed data; χ^2^-values (DF = 2 for *I*. *glandulifera* or DF = 3 for all other species) and resulting *p*-values are given. Different letters resulting from a post-hoc Tukey’s HSD test mark significant different groups for *p* < 0.050. Note that in the boxplots the untransformed data are presented. Boxes represent the first and third quartiles, bands inside the boxes the median. Whiskers are restricted to the 1.5 interquartile ranges. Datapoints not included in the whiskers are depicted as dots. target species are abbreviated as follows: *Geum urbanum* (*Geum urb*), *Filipendula ulmaria* (*Fil ulm*), *Urtica dioica* (*Urt dio*), *Salix fragilis* (*Sal fra*), *Lepidium sativum* (*Lep sat*) and *Impatiens glanduifera* (*Imp gla*).

In the pot experiment with juveniles, target plants were grown solitarily, in intraspecific competition, in competition with *I*. *glandulifera* or they were treated with 2-MNQ (Fig 3). An overall linear mixed-effect model (χ^2^ = 255.6, df = 14, *N* = 150, *p* < 0.001) showed that the juveniles’ biomass depended on species (χ^2^ = 279.80, df = 3, *p* < 0.001) and treatment (χ^2^ = 217.92, df = 3, *p* < 0.001). The species responded quite similar to the treatments (interaction species * treatment:χ^2^ = 14.69, df = 8, *p* = 0.065). On average the growth of all species was lower when they were watered with 2-MNQ than in the control, but only significant with respect to the biomass of *U*. *dioica* and *I*. *glandulifera* showing a growth reduction of 51% and 46%, respectively. All species except *F*. *ulmaria* competed intraspecifically, resulting in 66% less biomass in the most affected species *U*. *dioica*. The competition with *I*. *glandulifera* had an even stronger effect than the intraspecific competition, except on the height of *U*. *dioica*. In competition with *I*. *glandulifera* biomass of *U*. *dioica* was reduced by 85% compared to the control; in the most affected species *G*. *urbanum* biomass was reduced by 89%. In *I*. *glandulifera* the biomass of the target plant was reduced by 65% in competition with its conspecifics, whereas the height was not affected.

### Comparison of the impact of *I*. *glandulifera* in all trials

**Fig 4:**
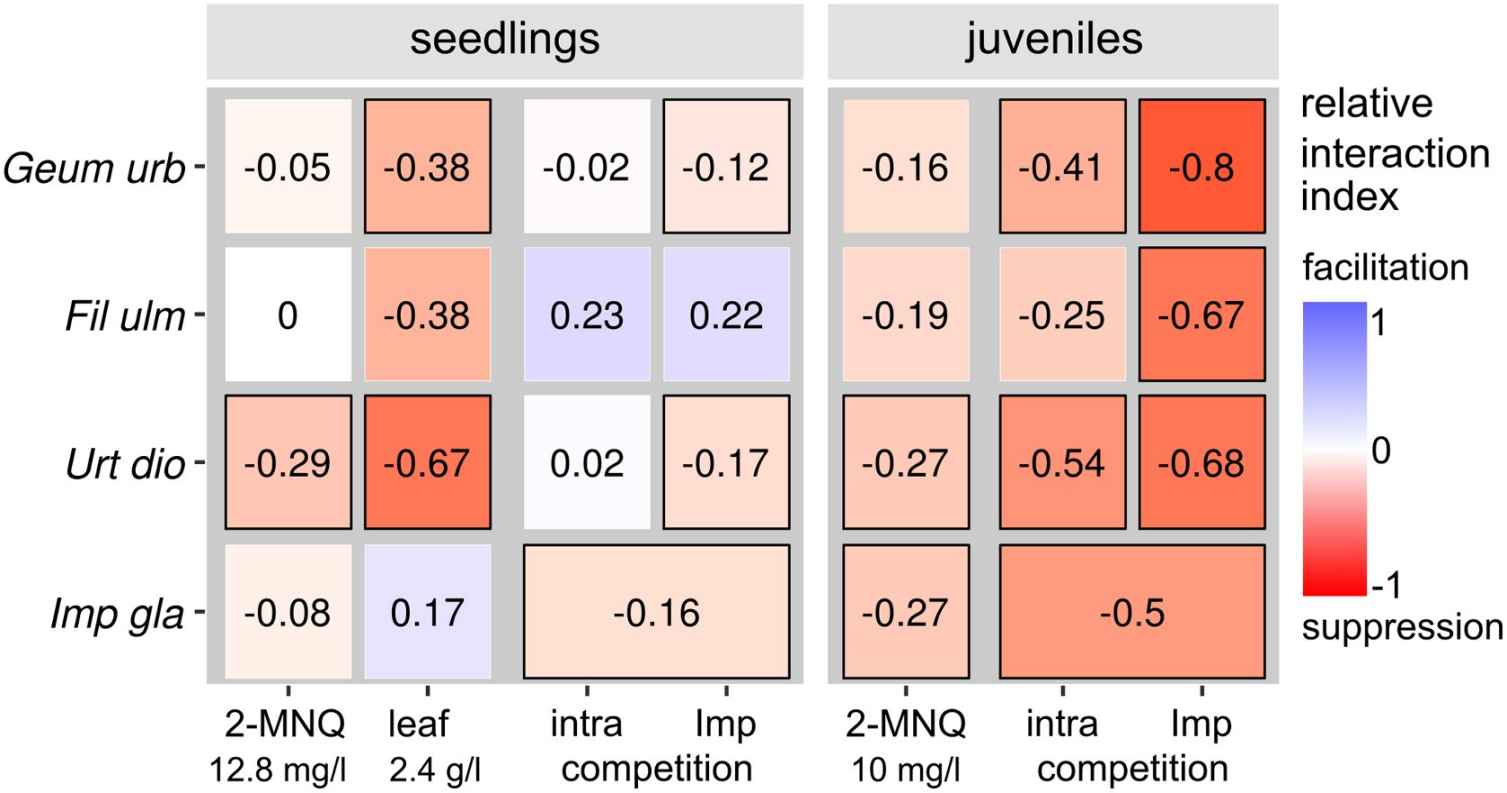
Intensity of the impact of competition and allelopathy by *I*. *glandulifera*. The intensity is expressed as relative interaction index among the different treatments and the appropriate control, calculated as (mean(treatment)-mean(control) / mean(treatment)+mean(control)). The relative interaction indices for all groups are given and represented by the colors of the heatmap. The more reddish the higher is a negative impact. Underlying growth parameters are root length of seedlings and aboveground dry biomass of juveniles. To visualize the impact of 2-MNQ and *I*. *glandulifera* leaf material (leaf) on seedlings the treatments with the highest concentrations were chosen. Black edged squares indicate that the growth of the target plants in the given treatment is significantly different from the related control. For the seedlings treated with 2-MNQ or *I*. *glandulifera* leaf material respectively, the black edged squares indicate an exponential decline of the root length in the bioassays. target species are abbreviated as follows: *Geum urbanum* (*Geum urb*), *Filipendula ulmaria* (*Fil ulm*), *Urtica dioica* (*Urt dio*), *Salix fragilis* (*Sal fra*), *Lepidium sativum* (*Lep sat*) and *Impatiens glanduifera* (*Imp gla*).

Negative relative interaction indices, as an indicator of the intensity of effects, showed that *I*. *glandulifera* allelochemicals and competition reduced the growth of the target species in all trials (Fig 4). The intensity of the impact depended on the species. *U*. *dioica* was most affected, considering the relative interaction indices as well as statistical differences between treatments and controls. In all cases, the growth of *U*. *dioica* was significantly reduced by *I*. *glandulifera*. *F*. *ulmaria* was the least affected as only the growth of juveniles in competition with *I*. *glandulifera* was significantly reduced. Furthermore, the impact depended on the developmental stage, with the juveniles being more affected than the seedlings, both by allelochemicals and competition. Hence, the relative interaction indices of intraspecific competition were lower in seedlings (up to -0.16 in *I*. *glandulifera*) than in the juveniles (up to -0.54 in *U*. *dioica*). Likewise, the competitive effect of *I*. *glandulifera* on seedlings was only expressed as a relative interaction index up to -0.17, whereas it was more than 4 times stronger on juveniles (relative interaction index ranging from -0.68 to -0.8). The effect of 2-MNQ was rather low as in the seedling trial it was lower than the effect of *I*. *glandulifera* leaf material. Similarly, in the experiment with juvenile plants it had a smaller impact than the competition with *I*. *glandulifera*. *I*. *glandulifera* seedlings were tolerant to 2-MNQ and *I*. *glandulifera* leaf material but juveniles were not (relative interaction index -0.27). Seedlings and juveniles of *I*. *glandulifera* competed intraspecifically, but the impact of *I*. *glandulifera* juveniles on their conspecifics was lower than on the native target species.

## Discussion

In the present study we compared impacts of *I*. *glandulifera* on different native plant species among seedlings and juvenile plants, in order to detect species-specific and developmental stage-specific effects. We found a competitive and allelopathic effect of *I*. *glandulifera* on target plants depending on species and developmental stage. Considering all trials *F*. *ulmaria* was the least and *U*. *dioica* the most affected species and in general the juveniles were more affected than the seedlings of all species.

### Dependency of competition and allelopathy on plant species and developmental stage

Species- and developmental stage-specific response to *I*. *glanduifera*, as we found, may be based on specific traits. In later developmental stages the studied target species change in their architecture. As juveniles *U*. *dioica*, *F*. *ulmaria* and *G*. *urbanum* start to build a rhizome from which they sprout in the following years. This could enable a fast growth in spring and give them an advantage in competition with *I*. *glandulifera* compared to plants developing from seeds. *U*. *dioica* however, seems to be sensitive to competition with *I*. *glandulifera* also when sprouting from rhizomes [5,53]. Furthermore, *F*. *ulmaria* initially forming a rosette can develop a flowering shoot from the second year onwards [54]. *F*. *ulmaria* may then reach a height of up to 2 m [36], which is comparable to *I*. *glandulifera* and could influence the outcome of their competition. Also comparing the species rosettes may be more shaded than tall growing plants, and hence affected by competition for light. In juveniles however we found no obvious difference among the response of the rosette forming species *F*. *ulmaria* and *G*. *urbanum* and stem building *U*. *dioica*. Nitrophilous species such as *U*. *dioica* may be more affected by competition for nutrients than competition for light. *I*. *glandulifera* can be considered as strong competitor due to its architecture. Tall plant growth is generally connected to a strong competitive effect, because tall plants shade co-occurring plants and remove other resources such as nutrients, water and space [55,56].

### Disentangling allelopathy from competition and the role of 2-MNQ

Effects of allelopathy and competition for resources are difficult to disentangle, because they interact with each other [57]. A possible method to detect allelopathy is to add activated charcoal to the plant substrate that absorbs allelopathic substances. Using this method [30] detected a rather large allelopathic impact of *I*. *glandulifera* on juvenile *U*. *dioica* in addition to competition. We found a negative effect of *I*. *glandulifera* among seedlings but adding activated charcoal did not reduce this effect, suggesting only a competitive effect. 2-MNQ is assumed to be the major allelopathic substance of *I*. *glandulifera* [21]. We found a negative but overall small effect of 2-MNQ on the growth of native plants. In juvenile plants, the effect of competition with *I*. *glandulifera* was much higher than the effect of 2-MNQ. When they are grown together with *I*. *glandulifera* the negative effects can be mediated by both competition and allelopathy as *I*. *glandulifera* should consume resources but may also secrete allelopathic substances. An interaction of competition and allelopathy may amplify their single effects.

In our study 2-MNQ had a lower impact on seedling growth than *I*. *glandulifera* leaf material. High impact of leaf material may also be intensified by a changed osmotic potential of the agar. Nevertheless, the lower impact of 2-MNQ indicates that 2-MNQ may not be the only substance responsible for the allelopathic effect of *I*. *glandulifera*. Likewise, [58] found no correlation between the allelopathic effect of senescent *I*. *glandulifera* leaves and their 2-MNQ content. However, the 2-MNQ content in their study was very low compared to the study of [21] who showed a negative effect of *I*. *glandulifera* shoot extracts on seed germination with higher concentrations of 2-MNQ. Several other substances were detected in *I*. *glandulifera* such as the naphtoquinone 2-hydroxy-naphtoquinone, other phenolic compounds, steroids, several flavonoids, or essential oils [22,24,25,59–62], could also be allelochemicals. For example the steroid glanduliferins A and B were shown to have an in vitro cytostatic effect [59]. In addition, 2-MNQ may have other effects, indirectly favoring the invasiveness of *I*. *glandulifera*. It can for example suppress the growth of mycorrhizal fungi [21] or reduce mycorrhiza colonization of some native species in soils invaded by *I*. *glandulifera* [28,29,63]. Furthermore, there might be a link between allelopathy and herbivore resistance. Pure 2-MNQ might have the potential to inhibit the reproduction of insects [64]. [58] in fact found no correlation between herbivore leaf damage and 2-MNQ concentration in senescent *I*. *glandulifera* leaves but a negative correlation with the concentration of the glycoside form of 2-MNQ.

### Effect of *I*. *glandulifera* on conspecifics

*I*. *glandulifera* plants were not fully tolerant to their conspecifics. We suggest that the tolerance of *I*. *glandulifera* seedlings to their own chemicals could enable massive seedling recruitment. In the seedling stage *I*. *glandulifera* produces a huge amount of allelochemicals [21] that can suppress other plant species. Due to tolerance towards their own chemicals *I*. *glandulifera* can form a dense and monospecific carpet of seedlings (own observations). During further development intraspecific competition becomes stronger and *I*. *glandulifera* plants become intolerant to their own allelochemicals as we observed in our trial with *I*. *glandulifera* juveniles. Such an allelopathic self-inhibition (“autotoxicity”) seems to be paradox but is often observed [65,66]. Self-inhibition may just be a side-effect outweighed by the benefit of inhibition of other species, but it is also thought to play a role in population dynamics [65–67]. It may intensify density-dependent mortality (“self-thinning”), and thus lead to spacing between individuals and reduce intraspecific resource competition among the remaining individuals. Autotoxicity should anyway not be a problem for species whose populations do not persist for long time on a specific site. These are, for example, species populations that are regularly replaced by succession [66]. It is known that crop plants can release allelopathic substances into the soil that impair the growth of their conspecifics in the following years [65]. Maybe autotoxicity can even induce the decline of a population. In the case of *I*. *glandulifera*, we suggest that autotoxicity of juveniles could intensify density-dependent mortality of individuals in *I*. *glandulifera* populations and play a role in the observed population fluctuations of this species [68]. Due to its high dispersal potential [5] *I*. *glandulifera* could compensate the collapse of a population by colonizing new sites rapidly.

### Consequences of *I*. *glandulifera* allelopathy and competition for native plant communities

In our study *I*. *glandulifera* overall suppressed the growth of the target species investigated. Therefore, we expect such a growth reduction also in the field. The response of our target species on *I*. *glandulifera* should be crucial for the native vegetation. Dominant species such as our target species are considered to make up a large portion of the community biomass and thereby determine the community structure [69]. For example, *F*. *ulmaria* plays a major role in succession dynamics. By suppressing other species it rapidly colonizes abandoned fields until it becomes senescent after several years and forest species are able to invade the area [54]. Here, *F*. *ulmaria* was the species least affected by *I*. *glandulifera* suggesting only a minor impact of *I*. *glandulifera* in associations dominated by *F*. *ulmaria*. Also *S*. *fragilis* that can form shrubs and start succession of woodlands after disturbances as flooding, was affected moderately. Suppression of *S*. *fragilis* by *I*. *glandulifera* may increase erosion as *S*. *fragilis* can protect riverside soil from erosion, whilst *I*. *glandulifera* is thought to favor erosion by not fixing the soil.

The fact that seedlings and juveniles as recruitment stages were affected should give *I*. *glandulifera* an advantage in cases where plant recruitment is crucial, i. e. when a plant colonizes new sites. Generally it can have important consequences on plant communities as early processes in plant development can determine community assembly [70]. So plant invasions can be enhanced by early superiority over native species (priority effect; [71,72]). The earlier a species is suppressed the more likely it should disappear from a community. *I*. *glandulifera* extracts and litter can reduce seed germination, the earliest step of plant recruitment, species-specifically [21,26,27]. This may have a more severe impact on the further development than the growth reduction of juvenile plants, as we have observed. All of our juvenile plants survived, hence they should also be able to establish. In established vegetation not only the competition ability of the plants, may be different compared to the early developmental stages, but also the importance of the components of competition may change. In the early stage of an invasion the ability of an invader to suppress natives is important. For the long-term success of an invasion in an established vegetation however, the ability of the invader to withstand competition by natives becomes more important (competitive-effect versus -response; [30,73]).

Species-specific sensitivity to *I*. *glandulifera* may lead to an altered community composition in the field with some species being more suppressed than others. Nevertheless, several field studies revealed only an overall weak effect of *I*. *glandulifera* on mostly riparian [18,19,74] and forest plant community composition and diversity [10]. As [18] discuss, this may be due to the fact that *I*. *glandulifera* just takes over the role of native dominant species and reduces their growth while species in the undergrowth remain unaffected. Likewise, we found that the competitive effect of *I*. *glandulifera* was in a comparable order of magnitude as the competitive effect of the natives on their conspecifics (intraspecific competition). In contrast to the afore-mentioned field studies [16,17] found a rather negative impact of *I*. *glandulifera* on riparian vegetation. Such ambiguities may be explained by different study conditions leading to different results due to additional context-dependencies. The consequence of competition between two species for a plant community is very complex and depends on several factors such as abiotic stress or the indirect reactions of other species [35]. Therefore, the impact of an invasive species on native communities should depend strongly on environment and ecosystem conditions such as climate, abiotic factors and the resident community [4].

## Conclusion

We conclude that the strong competitive effect of juvenile *I*. *glandulifera* should be caused by a combination of resource competition and allelopathic substances released by *I*. *glandulifera*. The low effect of 2-MNQ compared to *I*. *glandulifera* leaf material indicates that there could be allelopathic substances in addition to 2-MNQ. *I*. *glandulifera* was not fully tolerant to its conspecifics which may be connected to dynamics of *I*. *glandulifera* populations. Autotoxicity may intensify density-dependent mortality and eventually cause the known population fluctuations. We suggest that *I*. *glandulifera* reduces the growth of the native vegetation in the field. Species-specific growth reduction alters community composition with some species suppressed and others not. The succession of native plants might be delayed or changed but not fully hindered by *I*. *glandulifera*.

## Supporting information

S1 Fig Dependence of seedlings growth on the concentration of 2-MNQ. For each target species the root length, total dry biomass of the seedlings as well as number of seedlings that died shortly after placing the germinated seeds on the agar (no further growth observed) are shown. Using a linear model the regression equation f(x) = exp(*a*x + *b*) was fitted to test the dependency of root length and seedling biomass of the leaf material concentration; resulting *p*-values and coefficients *a* and *b* are given. Note that in contrast to Tab. 1 in results a linear model instead of a linear mixed effect model was used because the effect of random factor can not be visualized correctly with a regression line. target species are abbreviated as follows: *Geum urbanum* (*Geum urb*), *Filipendula ulmaria* (*Fil ulm*), *Urtica dioica* (*Urt dio*), *Salix fragilis* (*Sal fra*), *Lepidium sativum* (*Lep sat*) and *Impatiens glanduifera* (*Imp gla*).
(PDF)

S2 Fig Dependence of seedlings growth on the amount of *I*. *glandulifera* leaf material. For each target species the root length, total dry biomass of the seedlings as well as number of seedlings that died shortly after placing the germinated seeds on the agar (no further growth observed) are shown. Using a linear model the regression equation f(x) = exp(*a*x + *b*) was fitted to test the dependency of root length and seedling biomass on the amount of leaf material; resulting *p*-values and coefficients *a* and *b* are given. Note that in contrast to Tab. 1 in results a linear model instead of a linear mixed effect model was used because the effect of random factor can not be visualized correctly with a regression line. target species are abbreviated as follows: *Geum urbanum* (*Geum urb*), *Filipendula ulmaria* (*Fil ulm*), *Urtica dioica* (*Urt dio*), *Salix fragilis* (*Sal fra*), *Lepidium sativum* (*Lep sat*) and *Impatiens glanduifera* (*Imp gla*).
(PDF)

S1 Data Dataset of the seedling competition trial and allelopathy via roots. Seedlings of six target species were grown in five treatments testing competition and allelopathy of *I*. *glandulifera*. This dataset contains measured radicle length and biomass of the target seedlings dependent on the treatments. A description of all columns and factor levels is included in the document.
(TXT)

S2 Data Dataset of the seedling bioassay. Seedlings of six target species were treated with *I*. *glandulifera* leaf material or 2-MNQ to test the allelopathic effect of this substances. This dataset contains measured radicle length and biomass of the target seedlings dependent on the treatments. A description of all columns and factor levels is included in the document.
(TXT)

S3 Data Dataset of the juvenile trial. Juvenile plants of four target species were grown in four treatments testing competition and allelopathy of *I*. *glandulifera*. This dataset contains measured rosette projection area and biomass of the target plants dependent on the treatments. A description of all columns and factor levels is included in the document.
(TXT)

## References

[1] Sala OE, Chapin FS, Armesto JJ, Berlow E, Bloomfield J, Dirzo R, et al Global biodiversity scenarios for the year 2100. Science (80-). 2000;287(5459):1770–4. 10.1126/science.287.5459.1770 10710299

[2] Simberloff D. How common are invasion-induced ecosystem impacts? Biol Invasions. 2011;13(5):1255–68. 10.1007/s10530-011-9956-3

[3] Dogra KS, Sood SK, Dobhal PK, Sharma S. Alien plant invasion and their impact on indigenous species diversity at global scale: a review. J Ecol Nat Environ. 2010;2(9):175–86.

[4] Kueffer C, Pyšek P, Richardson DM. Integrative invasion science: model systems, multi‐site studies, focused meta‐analysis and invasion syndromes. New Phytol. 2013;200(3):615–33. 10.1111/nph.12415 23879193

[5] Beerling DJ, Perrins J. *Impatiens glandulifera* Royle (*Impatiens roylei* Walp.). J Ecol. 1993;81(2):367–82. 10.2307/2261507

[6] Perrins J, Fitter A, Williamson M. Population biology and rates of invasion of three introduced *Impatiens* species in the British Isles. J Biogeogr. 1993;20(1):33–44. 10.2307/2845737

[7] Pyšek P, Prach K. Invasion dynamics of *Impatiens glandulifera*—a century of spreading reconstructed. Biol Conserv. 1995;74(1):41–8. 10.1016/0006-3207(95)00013-T

[8] Malíková L, Prach K. Spread of alien *Impatiens glandulifera* along rivers invaded at different times. Ecohydrol Hydrobiol. 2010;10(1):81–5. 10.2478/v10104-009-0050-8

[9] Pyšek P, Prach K. Plant invasions and the role of riparian habitats: a comparison of four species alien to Central Europe. J Biogeogr. 1993;20(4):413–20. 10.2307/2845589

[10] Čuda J, Vítková M, Albrechtová M, Guo WY, Barney JN, Pyšek P. Invasive herb *Impatiens glandulifera* has minimal impact on multiple components of temperate forest ecosystem function. Biol Invasions. 2017;19(10):3051–66. 10.1007/s10530-017-1508-z

[11] Čuda J, Rumlerová Z, Brůna J, Skálová H, Pyšek P. Floods affect the abundance of invasive *Impatiens glandulifera* and its spread from river corridors. Divers Distrib. 2017;23(4):342–54. 10.1111/ddi.12524

[12] European Commission. Commission implementing regulation (EU) 2017/1263—of 12 July 2017—updating the list of invasive alien species of Union concern established by implementing regulation (EU) 2016/1141 pursuant to regulation (EU) No 1143/2014 of the European Parliament. Off J Eur Union. 2017;L 182:37–9.

[13] European Union. Invasive alien species of Union concern Luxembourg: Publications Office of the European Union; 2017 36 p.

[14] Info Flora. The national data and information center on the Swiss flora [Internet]. 2014 [cited 2018 Feb 26]. https://www.infoflora.ch/en/neophytes/lists.html

[15] Nehring S, Kowarik I, Rabitsch W, Essl F. Naturschutzfachliche Invasivitätsbewertungen für in Deutschland wild lebende gebietsfremde Gefäßpflanzen—BfN-Skripten 352 Nehring S, Kowarik I, Rabitsch W, Essl F, editors. Bonn: Bundesamt für Naturschutz; 2013 247 p.

[16] Cockel CP, Gurnell AM, Gurnell J. Consequences of the physical management of an invasive alien plant for riparian plant species richness and diversity. River Res Appl. 2014;30(2):217–29. 10.1002/rra.2633

[17] Hulme PE, Bremner ET. Assessing the impact of *Impatiens glandulifera* on riparian habitats: partitioning diversity components following species removal. J Appl Ecol. 2006;43(1):43–50. 10.1111/j.1365-2664.2005.01102.x

[18] Hejda M, Pyšek P. What is the impact of *Impatiens glandulifera* on species diversity of invaded riparian vegetation? Biol Conserv. 2006;132(2):143–52. 10.1016/j.biocon.2006.03.025

[19] Hejda M, Pyšek P, Jarošík V. Impact of invasive plants on the species richness, diversity and composition of invaded communities. J Ecol. 2009;97(3):393–403. 10.1111/j.1365-2745.2009.01480.x

[20] Levine JM, Vilà M, D’Antonio CM, Dukes JS, Grigulis K, Lavorel S. Mechanisms underlying the impacts of exotic plant invasions. Proc R Soc Ser B Biol Sci. 2003;270(1517):775–81. 10.1098/rspb.2003.2327 12737654 PMC1691311

[21] Ruckli R, Hesse K, Glauser G, Rusterholz H-P, Baur B. Inhibitory potential of naphthoquinones leached from leaves and exuded from roots of the invasive plant *Impatiens glandulifera*. J Chem Ecol. 2014;40(4):371–8. 10.1007/s10886-014-0421-5 24722883

[22] Bohm BA, Towers GHN. A study of phenolic compounds in *Impatiens*. Can J Bot. 1962;40(5):677–83. 10.1139/b62-065

[23] Chapelle J-P. 2-methoxy-1,4-naphthoquinone in *Impatiens glandulifera* and related species. Phytochemistry. 1974;13(3):662 10.1016/S0031-9422(00)91379-7

[24] Lobstein A, Brenne X, Feist E, Metz N, Weniger B, Anton R. Quantitative determination of naphthoquinones of *Impatiens* species. Phytochem Anal. 2001;12(3):202–5. 10.1002/pca.574 11705027

[25] Tříska J, Vrchotová N, Sýkora J, Moos M. Separation and identification of 1,2,4-trihydroxynaphthalene-1-O-glucoside in *Impatiens glandulifera* Royle. Molecules. 2013;18(7):8429–39. 10.3390/molecules18078429 23867652 PMC6269834

[26] Vrchotová N, Šerá B, Krejčová J. Allelopathic activity of extracts from *Impatiens* species. Plant, Soil Environ. 2011;57(2):57–60.

[27] Loydi A, Donath TW, Eckstein RL, Otte A. Non-native species litter reduces germination and growth of resident forbs and grasses: allelopathic, osmotic or mechanical effects? Biol Invasions. 2015;17(2):581–95. 10.1007/s10530-014-0750-x

[28] Ruckli R, Rusterholz H-P, Baur B. Invasion of an annual exotic plant into deciduous forests suppresses arbuscular mycorrhiza symbiosis and reduces performance of sycamore maple saplings. For Ecol Manage. 2014;318:285–93. 10.1016/j.foreco.2014.01.015

[29] Ruckli R, Rusterholz H-P, Baur B. Disrupting ectomycorrhizal symbiosis: indirect effects of an annual invasive plant on growth and survival of beech (*Fagus sylvatica*) saplings. Perspect Plant Ecol Evol Syst. 2016;19:12–20. 10.1016/j.ppees.2016.01.005

[30] Gruntman M, Pehl AK, Joshi S, Tielbörger K. Competitive dominance of the invasive plant Impatiens glandulifera: using competitive effect and response with a vigorous neighbour. Biol Invasions. 2014;16(1):141–51. 10.1007/s10530-013-0509-9

[31] Skálová H, Jarošík V, Dvořáčková, Pyšek P. Effect of intra- and interspecific competition on the performance of native and invasive species of *Impatiens* under varying levels of shade and moisture. PLoS One. 2013;8(5):e62842 10.1371/journal.pone.0062842 23675432 PMC3651240

[32] Čuda J, Skálová H, Janovský Z, Pyšek P. Competition among native and invasive *Impatiens* species: the roles of environmental factors, population density and life stage. AoB Plants. 2015;7:plv033-plv033. 10.1093/aobpla/plv033 25832103 PMC4417208

[33] Del Fabbro C, Güsewell S, Prati D. Allelopathic effects of three plant invaders on germination of native species: a field study. Biol Invasions. 2014;16(5):1035–42. 10.1007/s10530-013-0555-3

[34] Ammer C, Schall P, Wördehoff R, Lamatsch K, Bachmann M. Does tree seedling growth and survival require weeding of Himalayan balsam (*Impatiens glandulifera*)? Eur J For Res. 2011;130(1):107–16. 10.1007/s10342-010-0413-0

[35] Callaway RM, Walker LR. Competition and facilitation: a synthetic approach to interactions in plant communities. Ecology. 1997;78(7):1958–65. 10.1890/0012-9658(1997)078[1958:CAFASA]2.0.CO;2

[36] Hegi G. Illustrierte Flora von Mitteleuropa. Band IV, Teil 2A Conert H, Jäger E, Kadereit J, Schultze-Motel W, Wagenitz G, Weber H, editors. Berlin: Blackwell Wissenschafts-Verlag; 1995 704 p.

[37] Ellenberg H, Leuschner C. Vegetation Mitteleuropas mit den Alpen. Stuttgart: Ulmer; 2010 1334 p.

[38] Quinger B. *Salix fragilis* L. In: Sebald O, Seybold S, Philippi G, editors. Die Farn- und Bl00FCtenpflanzen Baden-Württembergs, Band 2. Stuttgart: Ulmer; 1990 p. 135–8.

[39] Macías FA, Castellano D, Molinillo JMG. Search for a standard phytotoxic bioassay for allelochemicals. Selection of standard target species. J Agric Food Chem. 2000;48(6):2512–21. 10.1021/jf9903051 10888578

[40] Inderjit, Callaway RM. Experimental designs for the study of allelopathy. Plant Soil. 2003;256(1):1–11. 10.1023/A:1026242418333

[41] Fujii Y, Shibuya T, Nakatani K, Itani T, Hiradate S, Parvez MM. Assessment method for allelopathic effect from leaf litter leachates. Weed Biol Manag. 2004;4(1):19–23. 10.1111/j.1445-6664.2003.00113.x

[42] RCoreTeam. R: a language and environment for statistical computing [Internet]. R Foundation for Statistical Computing; 2017 [cited 2018 Feb 5]. https://www.r-project.org/

[43] Bates D, Maechler M, Bolker B, Walker S. Fitting linear mixed-effects models using lme4. J Stat Softw. 2015;67(1):1–48. doi: 10.18637/jss.v067.i01

[44] Wickham H. ggplot2: elegant graphics for data analysis. New York: Springer; 2009. 213 p.

[45] Wickham H. The split-apply-combine strategy for data analysis. J Stat Softw. 2011;40(1):1–29. doi: 10.18637/jss.v040.i01

[46] Hothorn T, Bretz F, Westfall P. Simultaneous inference in general parametric models. Biometrical J. 2008;50(3):346–63. 10.1002/bimj.200810425 18481363

[47] Wilke CO. cowplot: streamlined plot theme and plot annotations for “ggplot2” [Internet]. 2017 [cited 2018 Feb 5]. https://cran.r-project.org/package=cowplot

[48] Neuwirth E. RColorBrewer: color brewer palettes [Internet]. 2014 [cited 2018 Feb 5]. https://cran.r-project.org/package=RColorBrewer

[49] Robinson D. broom: convert statistical analysis objects into tidy data frames [Internet]. 2017 [cited 2018 Feb 5]. https://cran.r-project.org/package=broom

[50] Hope RM. Rmisc: ryan miscellaneous [Internet]. 2013 [cited 2018 Mar 8]. https://cran.r-project.org/package=Rmisc

[51] Fox J, Weisberg S. An R companion to applied regression. Thousand Oaks: Sage; 2011 449 p.

[52] Armas C, Ordiales R, Pugnaire FI. Measuring plant interactions: a new comparative index. Ecology. 2004;85(10):2682–6. 10.1890/03-0650

[53] Tickner DP, Angold PG, Gurnell AM, Mountford JO, Sparks T. Hydrology as an influence on invasion: experimental investigations into competition between the alien *Impatiens glandulifera* and the native *Urtica dioica* in the UK In: Brundu G, Brock J, Camarda I, Child L, Wade M, editors. Plant invasions: species ecology and ecosystem management. Leiden: Blackhuys Publishers; 2001 p. 159–68.

[54] Falinska K. Genet disintegration in *Filipendula ulmaria*: Consequences for population dynamics and vegetation succession. J Ecol. 1995;83(1):9–21. 10.2307/2261146

[55] Goldberg D. Components of resource competition in plant communities In: Grace JB, Tilman D, editors. Perspectives on plant competition. San Diego: Academic Press; 1990 p. 27–49.

[56] Grime JP. Evidence for the existence of three primary strategies in plants and its relevance to ecological and evolutionary theory. Am Nat. 1977;111(982):1169–94.

[57] Inderjit, Moral R. Is separating resource competition from allelopathy realistic? Bot Rev. 1997;63(3):221–30. 10.1007/BF02857949

[58] Gruntman M, Segev U, Glauser G, Tielbörger K. Evolution of plant defences along an invasion chronosequence: defence is lost due to enemy release—but not forever. J Ecol. 2017;105(1):255–64. 10.1111/1365-2745.12660

[59] Cimmino A, Mathieu V, Evidente M, Ferderin M, Moreno L, Banuls Y, et al Glanduliferins A and B, two new glucosylated steroids from *Impatiens glandulifera*, with in vitro growth inhibitory activity in human cancer cells. Fitoterapia. 2016;109:138–45. 10.1016/j.fitote.2015.12.016 26732071

[60] Szewczyk K, Zidorn C, Biernasiuk A, Komsta Ł, Granica S. Polyphenols from *Impatiens* (Balsaminaceae) and their antioxidant and antimicrobial activities. Ind Crops Prod. 2016;86:262–72. 10.1016/j.indcrop.2016.03.053

[61] Szewczyk K, Kalemba D, Komsta Ł, Nowak R. Comparison of the essential oil composition of selected *Impatiens* species and its antioxidant activities. Molecules. 2016;21(9):1162 10.3390/molecules21091162 27598111 PMC6274178

[62] Vieira MN, Winterhalter P, Jerz G. Flavonoids from the flowers of *Impatiens glandulifera* Royle isolated by high performance countercurrent chromatography. Phytochem Anal. 2016;27(2):116–25. 10.1002/pca.2606 26751603

[63] Tanner RA, Gange AC. The impact of two non-native plant species on native flora performance: potential implications for habitat restoration. Plant Ecol. 2013;214(3):423–32. 10.1007/s11258-013-0179-9

[64] Mitchell MJ, Brescia AI, Smith SL, Morgan ED. Effects of the compounds 2-methoxynaphthoquinone, 2-propoxynaphthoquinone, and 2-isopropoxy-naphthoquinone on ecdysone 20-monooxygenase activity. Arch Insect Biochem Physiol. 2007;66(1):45–52. 10.1002/arch.20196 17694563

[65] Singh HP, Batish DR, Kohli RK. Autotoxicity: concept, organisms, and ecological significance. Vol. 18, Critical Reviews in Plant Sciences. 1999 p. 757–72.

[66] Whittaker RH, Feeny PP. Allelochemics: chemical interactions between species. Science (80-). 1971;171(3973):757–70. 10.1126/science.171.3973.757 5541160

[67] Canals RM, Emeterio LS, Peralta J. Autotoxicity in *Lolium rigidum*: analyzing the role of chemically mediated interactions in annual plant populations. J Theor Biol. 2005;235(3):402–7. 10.1016/j.jtbi.2005.01.020 15882702

[68] Kasperek G. Fluctuations in numbers of neophytes, especially *Impatiens glandulifera*, in permanent plots in a west German floodplain during 13 years. In: Kühn I, Klotz S, editors. Biological invasions Challenges for science—Neobiota 3. 2004 p. 27–37.

[69] Gioria M, Osborne BA. Resource competition in plant invasions: emerging patterns and research needs. Front Plant Sci. 2014;5:501 10.3389/fpls.2014.00501 25324851 PMC4179379

[70] Fukami T. Historical contingency in community assembly: integrating niches, species pools, and priority effects. Annu Rev Ecol Evol Syst. 2015;46:1–23. 10.1146/annurev-ecolsys-110411-160340

[71] Gioria M, Pyšek P, Osborne BA. Timing is everything: Does early and late germination favor invasions by herbaceous alien plants? J Plant Ecol. 2018;11(1):4–16. 10.1093/jpe/rtw105

[72] Goodale KM, Wilsey BJ. Priority effects are affected by precipitation variability and are stronger in exotic than native grassland species. Plant Ecol. 2018;219(4):429–39. 10.1007/s11258-018-0806-6

[73] Hager HA. Competitive effect versus competitive response of invasive and native wetland plant species. Oecologia. 2004;139(1):140–9. 10.1007/s00442-004-1494-6 14758534

[74] Diekmann M, Effertz H, Baranowski M, Dupré C. Weak effects on plant diversity of two invasive *Impatiens* species. Plant Ecol. 2016;217:1503–14. 10.1007/s11258-016-0663-0

