# Supplementary figures and images for: Species- and developmental stage-specific effects of allelopathy and competition of invasive Impatiens glandulifera on co-occurring plants
Source: PLoS One. 2018 Nov 7;13(11):e0205843. doi: 10.1371/journal.pone.0205843 (PMC6221290; doi:10.1371/journal.pone.0205843)

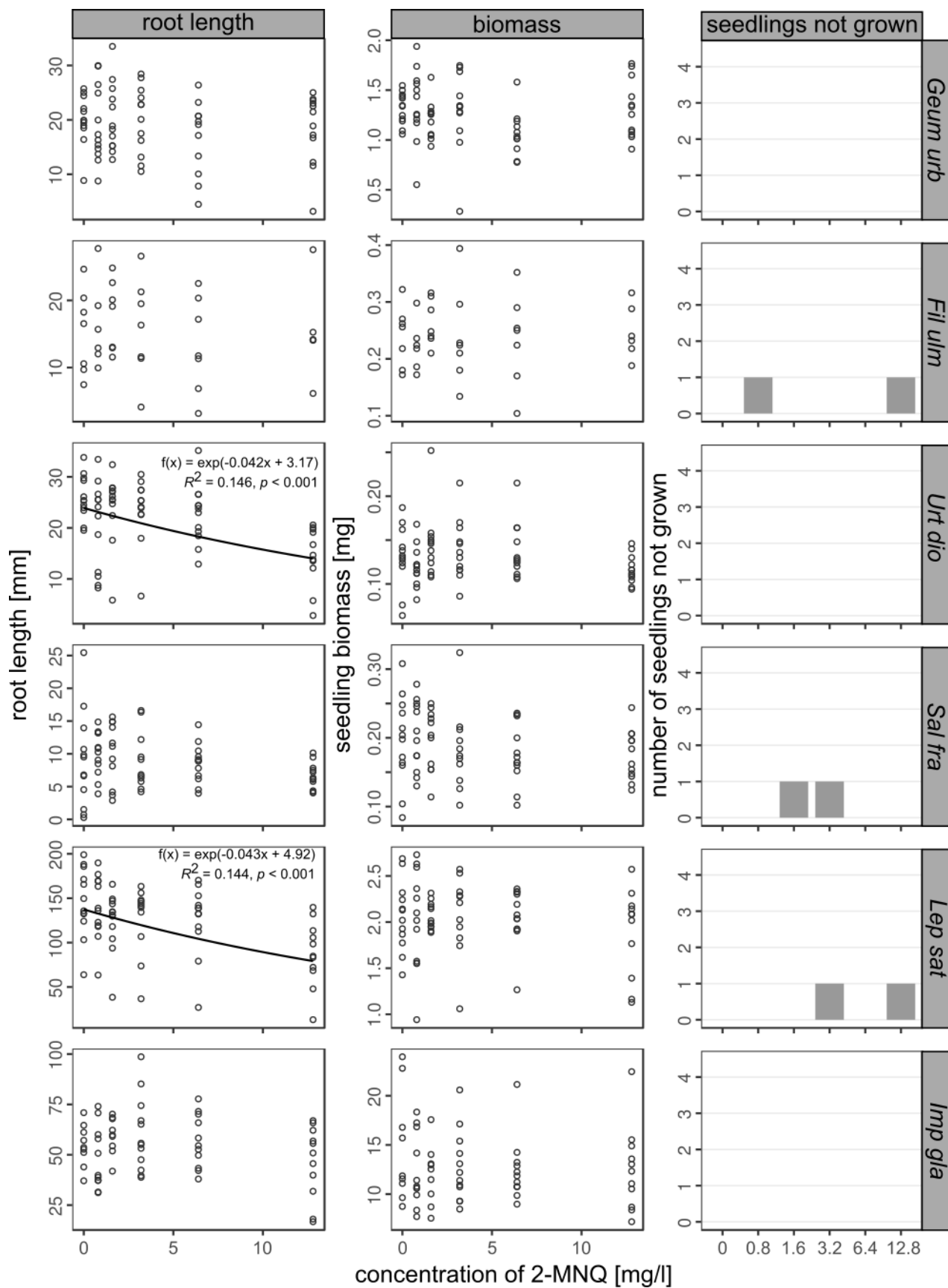

S1 Fig. Dependence of seedlings growth on the concentration of 2-MNQ.

Supplement: S1 Fig — For each target species the root length, total dry biomass of the seedlings as well as number of seedlings that died shortly after placing the germinated seeds on the agar (no further growth observed) are shown. Using a linear model the regression equation f(x) = exp(ax + b) was fitted to test the dependency of root length and seedling biomass of the leaf material concentration; resulting p-values and coefficients a and b are given. Note that in contrast to Tab. 1 in results a linear model instead of a linear mixed effect model was used because the effect of random factor can not be visualized correctly with a regression line. target species are abbreviated as follows: Geum urbanum (Geum urb), Filipendula ulmaria (Fil ulm), Urtica dioica (Urt dio), Salix fragilis (Sal fra), Lepidium sativum (Lep sat) and Impatiens glanduifera (Imp gla). (PDF) [file pone.0205843.s001.pdf]

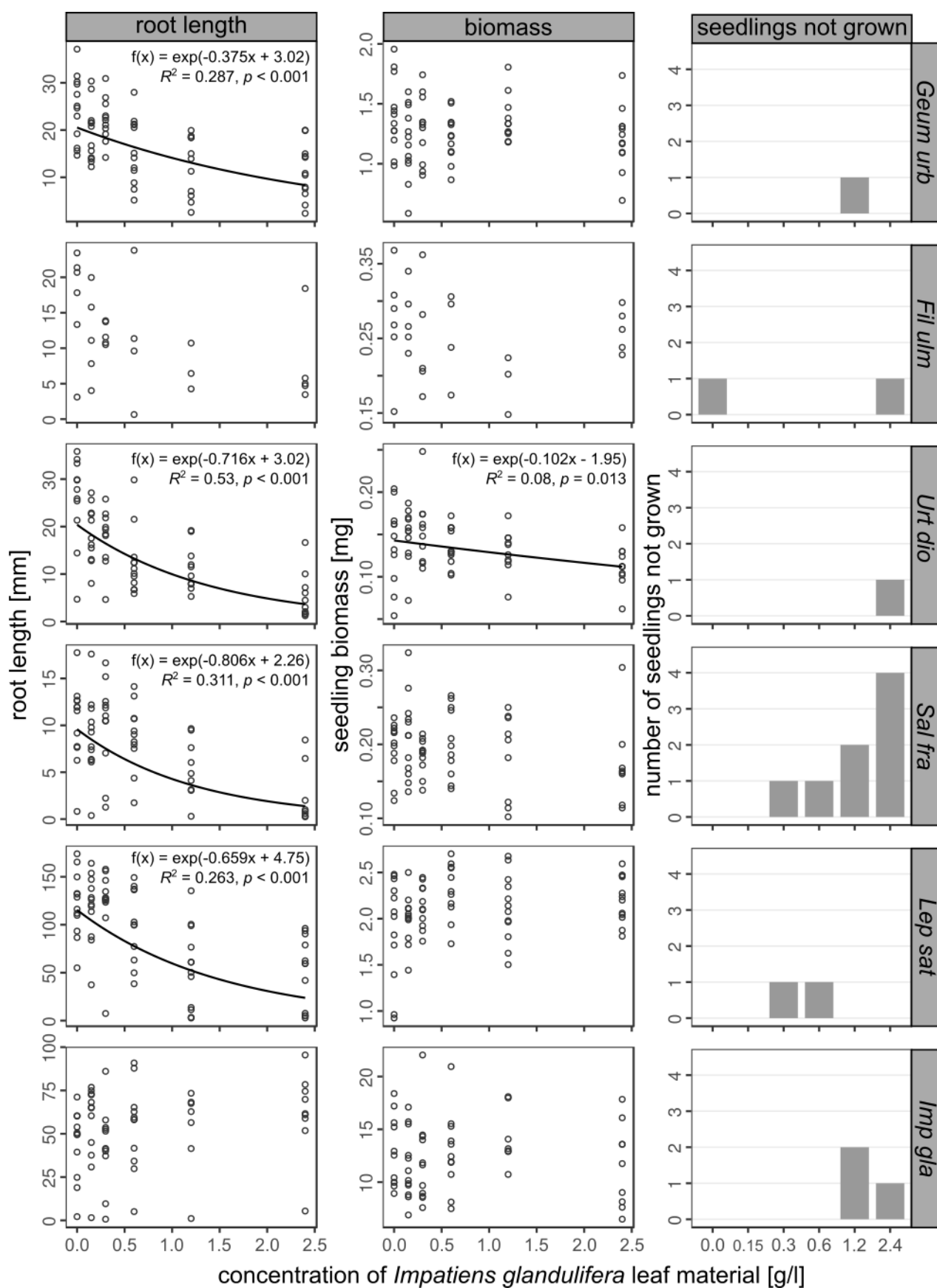

S2 Fig. Dependence of seedlings growth on the amount of *I. glandulifera* leaf material.

Supplement: S2 Fig — For each target species the root length, total dry biomass of the seedlings as well as number of seedlings that died shortly after placing the germinated seeds on the agar (no further growth observed) are shown. Using a linear model the regression equation f(x) = exp(ax + b) was fitted to test the dependency of root length and seedling biomass on the amount of leaf material; resulting p-values and coefficients a and b are given. Note that in contrast to Tab. 1 in results a linear model instead of a linear mixed effect model was used because the effect of random factor can not be visualized correctly with a regression line. target species are abbreviated as follows: Geum urbanum (Geum urb), Filipendula ulmaria (Fil ulm), Urtica dioica (Urt dio), Salix fragilis (Sal fra), Lepidium sativum (Lep sat) and Impatiens glanduifera (Imp gla). (PDF) [file pone.0205843.s002.pdf]
